# Successful therapy with bevacizumab combined with corticosteroids for crizotinib-induced interstitial lung disease

**DOI:** 10.1007/s10456-019-09673-1

**Published:** 2019-06-27

**Authors:** Xiaohong Xie, Bingpeng Guo, Xinqing Lin, Yinyin Qin, Ming Ouyang, Shiyue Li, Chengzhi Zhou

**Affiliations:** State Key Laboratory of Respiratory Disease, National Clinical Research Center for Respiratory Disease, Guangzhou Institute of Respiratory Health, The First Affiliated Hospital of Guangzhou Medical University, No. 151 Yanjiang Road, Guangzhou, 510120 Guangdong People’s Republic of China

**Keywords:** Crizotinib, Anaplastic lymphoma kinase, Interstitial lung disease, Bevacizumab

## Abstract

We present the case of an old woman with ALK-rearranged stage IV lung adenocarcinoma who received crizotinib. She presented with severe dyspnea on the 34th day, and diffuse ground-glass opacifications in her chest. A diagnosis of crizotinib-induced ILD was confirmed. Corticosteroids were administered. However, the disease was still progressing rapidly. Therefore, as a monoclonal antibody against vascular endothelial growth factor, bevacizumab was administered in low doses (200 mg on days one and three). Her symptoms began to improve. Our clinical experience indicates that bevacizumab combined with corticosteroids might be a promising treatment in crizotinib-induced ILD patients.

## Case

The patient was a 59-year-old woman with stage IVb lung adenocarcinoma T4N3M1c. 18F-fluorodeoxyglucose positron emission tomography/computed tomography (CT) revealed metastatic lesions involving the left lower lobe and left hilar, mediastinum, bilateral supraclavicular region, multiple retroperitoneal lymph nodes, and left pleura; multiple metastases in the liver and bone metastases of the entire body, and bone. The presence of EML4-AKL rearrangement was revealed through real-time reverse transcription-polymerase chain reaction. Crizotinib was administered twice daily at a dose of 250 mg. Twenty-one days after the initiation of crizotinib treatment, she showed no adverse effects, and there was a decrease in the neurogene-specific enolase value, anti-non-small-cell-lung cancer (NSCLC) in the serum. A comparison analysis of the whole-body PET/CT scans between before and after crizotinib treatment demonstrated a reduction of left lower lung lesions and pleural effusion. The efficacy evaluation of therapy reached PR.

**Fig. 1:**
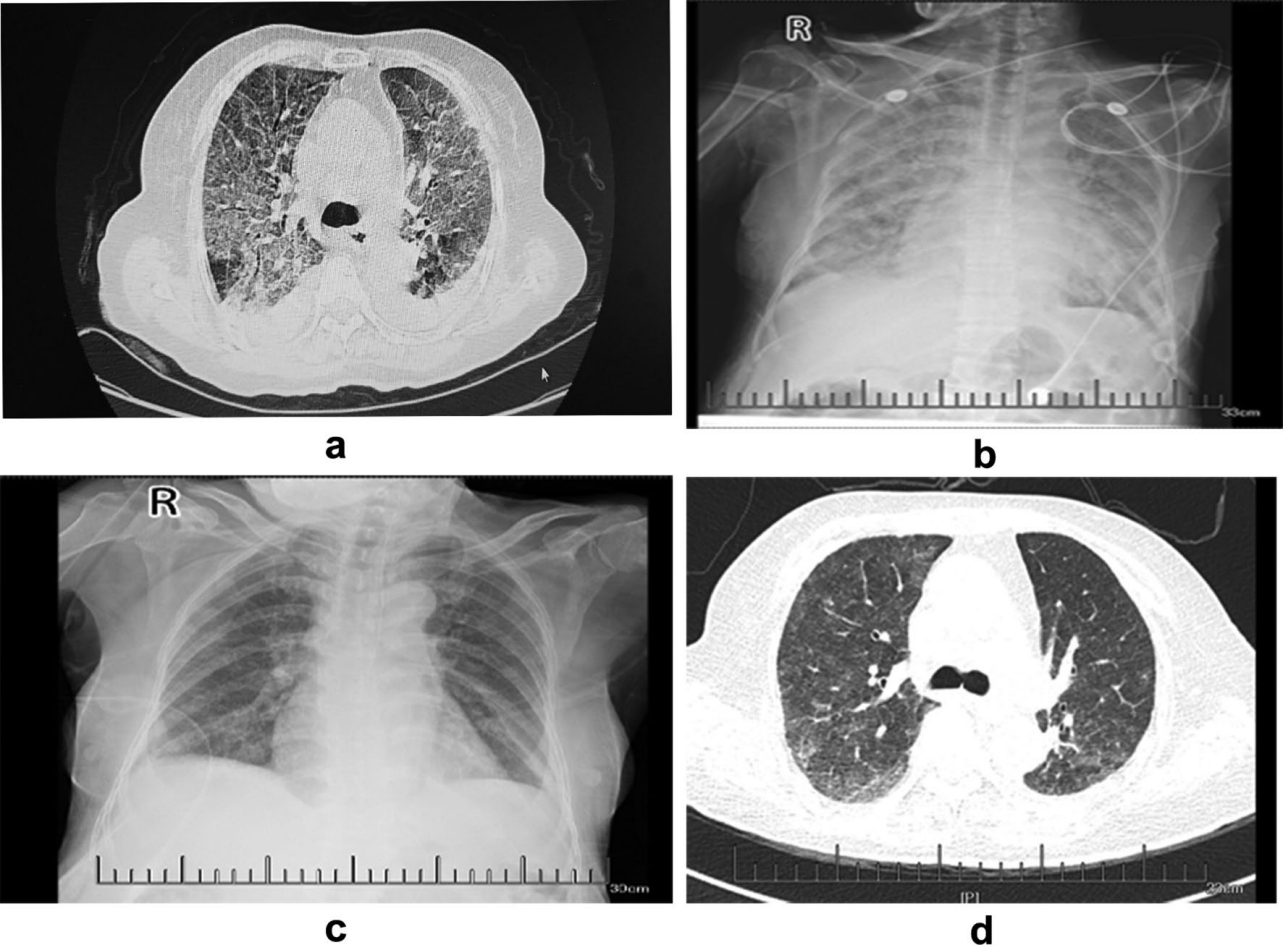
**a** On the 34th day following crizotinib treatment, the CT showed diffusing bilateral increased ground-glass opacity and reticulation. **b** Chest X-ray showing diffused bilateral ground-glass opacity. **c** Chest X-ray showing diffused bilateral ground-glass opacity after treatment with methylprednisolone and bevacizumab. **d** Chest CT scans showing that the diffuse lesions of both lungs were improved and the pleural effusion decreased significantly.

However, on the 34th day, she presented with aggravated dry cough and dyspnea and was subsequently admitted to a local hospital. A chest CT showed diffusing bilateral increased ground-glass opacity and reticulation and a medium amount of pleural effusion (Fig. 1a). A diagnosis of pneumonia and severe respiratory failure was made. Non-invasive positive pressure ventilation (NIPPV) and empirical antibiotics with meropenem and vancomycin were administered, but her progressive dyspnea and severe hypoxemia worsened. She was then transferred to our hospital. Her heart rate was 127 beats/min (bpm), and her respiratory rate was 32 breaths/min. Pulse oximetry revealed hypoxemia and 90% oxyhemoglobin saturation. The oxygen index (OI) value was 82.5, her leukocyte count was 13.2 × 10^9^/L, and her procalcitonin level was 2.28 ng/mL; no definite pathological microorganism was found in either the sputum or blood cultures. Troponin and pro-brain-type natriuretic peptide levels and echocardiography results were normal. A chest X-ray showed diffusing bilateral ground-glass opacity (Fig. 1b).

Although we were unable to evaluate the patient through bronchoscopy and lung biopsy because of her severe respiratory failure, a diagnosis of crizotinib-induced ILD, pneumonia, and type I respiratory failure was considered. The patient and her family members refused invasion ventilation. Crizotinib was discontinued, and NIPPV was supported with an inspired oxygen fraction (FiO_2_) of 80%. Treatment with 40 mg of methylprednisolone twice a day was initiated. However, the patient still had severe dyspnea. Then, 200 mg of bevacizumab was administered once a day on the second and fourth days, and her conditions gradually improved. There was a decrease in ground-glass opacity after treatment with methylprednisolone and bevacizumab, as shown by a chest X-ray (Fig. 1c).

Methylprednisolone was tapered to 32 mg daily and then reduced to 4 mg/d per week. As a second-line therapy, anlotinib was given orally five days later, once daily (8 mg) on days 1 to 7, which was increased to 12 mg daily on days 7 to 14 of a 21-day cycle. Chest CT scans showed that the diffuse lesions of both lungs were significantly absorbed, and the pleural effusion was significantly reduced seven days after anlotinib treatment (Fig. 1d).

On the 21st day after anlotinib initiation, the hilar and mediastinum regions and the bilateral ground-glass opacity decreased. Anlotinib (12 mg daily) was still given on days 1 to 14 of a 21-day cycle.

## Discussion

To the best of our knowledge, this is the first case of successful treatment with traditional steroids and an antiangiogenic monoclonal antibody, bevacizumab, in a patient diagnosed with crizotinib-induced ILD [1]. The incidence of ILD induced by crizotinib is 1.2% overall and 3.7% in the Japanese population [2]. The median age of the ILD group was 58.5 years, occurred at any time during the course of crizotinib therapy, but the median onset of crizotinib-induced ILD was 23 days. The mortality rate of patients with crizotinib-induced ILD is 50% [2].

Current treatment management includes corticosteroids. However, most individuals with severe disease were treated with high-dose corticosteroids (1 g/d intravenously), but adverse effects such as infection or hemorrhage of the gastrointestinal mucosa could aggravate the disease.

Bevacizumab is a recombinant humanized monoclonal antibody against VEGFR that causes a significant extension in overall survival and progression-free survival. When combined with paclitaxel and carboplatin, it improves the overall response rate of patients with advanced non-squamous NSCLC [3]. In patients with ILD, neovascularization is a fundamental process required for the recovery of lung interstitial tissue after lung injury; this process is affected by VEGF and others. Nintedanib, which also inhibits the receptors of VEGF, as well as platelet-derived growth factors (PDGFs) and fibroblast growth factors, showed a trend toward reducing declines in lung function, with fewer acute exacerbations and preserved quality of life [4]. Considering the relative contraindication of high-dose corticosteroids and the pharmacologic mechanism of bevacizumab, we considered treatment with methylprednisolone combined with bevacizumab. To our surprise, the patient responded well to this treatment, with improvements in dyspnea, OI, and ground-glass opacity according to the chest X-ray.

In clinical practice, a disease flare-up is a life-threatening condition, particularly after the discontinuation of molecular-targeted drugs. When a flare-up after drug-induced ILD is encountered, it is critical to apply an alternative treatment strategy. Anlotinib is an inhibitor that targets multiple receptor tyrosine kinases, particularly VEGFR, PDGFβ, and the stem cell-factor receptor (c-Kit). We considered anlotinib as the second-line therapy.

In conclusion, our case indicates that bevacizumab combined with corticosteroids could be a treatment for crizotinib-related ILD. In future works, we plan to accumulate more clinical experience and trials to confirm our results.
